# Impact of the integration of proton magnetic resonance imaging spectroscopy to PI-RADS 2 for prediction of high grade and high stage prostate cancer

**DOI:** 10.1590/0100-3984.2016.0117

**Published:** 2017

**Authors:** Michael S. Leapman, Zhen J. Wang, Spencer C. Behr, John Kurhanewicz, Ronald J. Zagoria, Peter R. Carroll, Antonio C. Westphalen

**Affiliations:** 1 MD, Department of Urology, University of California San Francisco, San Francisco, CA, USA.; 2 MD, Department of Radiology and Biomedical Imaging, University of California San Francisco, San Francisco, CA, USA.; 3 PhD, Department of Radiology and Biomedical Imaging, University of California San Francisco, San Francisco, CA, USA.; 4 MPH, MD, Department of Urology, University of California San Francisco, San Francisco, CA, USA.; 5 MD, PhD, Department of Radiology and Biomedical Imaging and Department of Urology, University of California San Francisco, San Francisco, CA, USA.

**Keywords:** MRI, Spectroscopy, Diagnosis, Prostate cancer, Prostatectomy, Espectroscopia, Diagnóstico, Câncer de próstata, Prostatectomia

## Abstract

**Objective:**

To compare the predictions of dominant Gleason pattern ≥ 4 or non-organ confined disease with Prostate Imaging Reporting and Data System (PI-RADS v2) with or without proton magnetic resonance spectroscopic imaging (^1^H-MRSI).

**Materials and Methods:**

Thirty-nine men underwent 3-tesla endorectal multiparametric MRI including ^1^H-MRSI and prostatectomy. Two radiologists assigned PI-RADS v2 and ^1^H-MRSI scores to index lesions. Statistical analyses used logistic regressions, receiver operating characteristic (ROC) curves, and 2x2 tables for diagnostic accuracies.

**Results:**

The sensitivity and specificity of ^1^H-MRSI and PI-RADS v2 for high-grade prostate cancer (PCa) were 85.7% (57.1%) and 92.9% (100%), and 56% (68.0%) and 24.0% (24.0%). The sensitivity and specificity of ^1^H-MRSI and PI-RADS v2 for extra-prostatic extension (EPE) were 64.0% (40%) and 20.0% (48%), and 50.0% (57.1%) and 71.4% (64.3%). The area under the ROC curves (AUC) for prediction of high-grade prostate cancer were 0.65 and 0.61 for PI-RADS v2 and 0.72 and 0.70 when combined with ^1^H-MRSI (readers 1 and 2, *p* = 0.04 and 0.21). For prediction of EPE the AUC were 0.54 and 0.60 for PI-RADS v2 and 0.55 and 0.61 when combined with ^1^H-MRSI (*p* > 0.05).

**Conclusion:**

^1^H-MRSI might improve the discrimination of high-grade prostate cancer when combined to PI-RADS v2, particularly for PI-RADS v2 score 4 lesions, but it does not affect the prediction of EPE.

## Abstract (pt)

**Objetivo:**

Comparar as predições de tumor com padrão 4 de Gleason dominante ou de tumor com extensão extraprostática utilizando o sistema *Prostate Imaging Reporting and Data System* (PI-RADS v2), combinado ou não a espectroscopia por ressonância magnética (^1^H-ERM).

**Materiais e Métodos:**

Trinta e nove pacientes submeteram-se a RM de 3 tesla com bobina endorretal, incluindo ^1^H-ERM, e prostatectomia. Dois radiologistas classificaram as principais lesões identificadas em cada caso utilizando PI-RADS v2 e escores de ^1^H-ERM. As análises estatísticas incluíram regressões logísticas, curvas *receiver operating characteristic* (ROC) e tabelas 2x2 para acurácia diagnóstica.

**Resultados:**

A sensibilidade e a especificidade da ^1^H-ERM e do PI-RADS v2 para a detecção de câncer de próstata de alto grau foram 85,7% (57,1%) e 92,9% (100%), e 56% (68%) e 24% (24%). A sensibilidade e a especificidade da ^1^H-ERM e do PI-RADS v2 para a detecção de extensão extraprostática (EEP) foram 64,0% (40%) e 20% (48%), e 50% (57,1%) e 71,4% (64,3%). As áreas das curvas ROC para a predição de câncer de alto grau foram 0,65 e 0,61 para PI-RADS v2 e 0,72 e 0,70 quando combinado com ^1^H-ERM (radiologistas 1 e 2, *p* = 0.04 e 0.21). Para a predição de EEP, as áreas das curvas ROC foram 0,54 e 0,60 para PI-RADS v2 e 0,55 e 0,61 quando combinado com ^1^H-ERM (*p* > 0.05).

**Conclusão:**

É possível que a ^1^H-ERM melhore a predição de câncer de alto grau quando combinada ao PI-RADS v2, em particular para lesões que recebem um escore PI-RADS v2 4; entretanto, ela não afeta a predição de EEP.

## INTRODUCTION

Prostate cancer (PCa) is diagnosed in approximately 230,000 men in the United States each year^(1)^, the majority of whom will possess favorable risk disease and in whom conservative approaches including active surveillance may be prudent^(2)^. Multiparametric magnetic resonance imaging (mpMRI) of the prostate has gained considerable utilization in the setting of newly diagnosed disease to identify occult, higher-grade or stage elements missed by conventional biopsy^(3,4)^. Moreover, when coupled with real time ultrasonography, fusion mpMRI biopsy has demonstrated superior PCa detection rates compared with traditional template guided biopsy^(5)^.

With growing integration of mpMRI as an adjunct diagnostic modality, the need to standardize acquisition protocols and study reporting is evident as it may facilitate benchmarks for consistency in both clinical care and research settings alike^(6)^. The American College of Radiology, the AdMeTech Foundation, and the European Society of Urogenital Radiology have partnered and recently presented a new version of the Prostate Imaging Reporting and Data System (PI-RADS v2), which integrates results of T2-weighted (T2W), high b-value diffusion-weighted image (DWI), and dynamic contrast enhanced (DCE) MRI^(7)^. Proton MR spectroscopic imaging (^1^H-MRSI), previously an optional tool, was not included in the current version of the document. ^1^H-MRSI has, though, been recognized as a useful non-invasive method for evaluating metabolic characteristics of prostatic lesions, yielding identifiable signatures that may allow for the discrimination of high-grade tumors^(8)^. However, ^1^H-MRSI is susceptible to false positive related to choline contamination from the seminal vesicles or urethra^(9)^, or by prostatitis^(10)^. Furthermore, the ACRIN 6659 study that was published by Weinreb et al. found no added benefit for ^1^H-MRSI compared with T2W alone to localize PCa to the gland sextant^(11)^.

In this context, we sought to compare the diagnostic performance of PI-RADS v2 with or without ^1^H-MRSI for predicting PCa with dominant Gleason pattern ≥ 4 or non-organ confined disease at the time of surgery.

## MATERIALS AND METHODS

The Institutional Review Board approved this retrospective single center study. Informed consent was prospectively obtained from all patients authorizing the use of clinical data in future studies. Consecutive subjects were identified through searches of our Urological Oncological Database, Prostate MR Imaging Database, and electronic medical records. Inclusion criteria: biopsy-proven PCa; 3-tesla endorectal prostate mpMRI, including ^1^H-MRSI; radical prostatectomy within six months of imaging; no treatments between imaging and surgery.

Forty patients seen between January 2013 and December 2014 fulfilled these criteria, but one was excluded because of a hip replacement that distorted the ^1^H-MRSI data. Therefore, 39 men formed the study population. Patients were clinically risk stratified using the Cancer of the Prostate Risk Assessment score (CAPRA)^(12)^. CAPRA is an easy to calculate validated nomogram that predicts outcomes across multiple treatment approaches and it predicts an individual’s likelihood of metastasis, cancer-specific mortality, and overall mortality. The score is calculated using points assigned to: age at diagnosis, PSA at diagnosis, Gleason score of the biopsy, clinical stage and percent of biopsy cores involved with cancer. Three categories were assigned: low (scores 0-2), intermediate (scores 3-5), and high risk (scores 6-10).

### MRI technique

Scans were acquired on a 3-tesla scanner (GE Healthcare, Waukesha, WI, USA) using the body coil for excitation and an endorectal coil (E-Coil; Medrad, Pittsburgh, PA, USA) filled with perfluorocarbon (Flutech_T14 TM; F2 Chemicals, UK) and a phased-array coil for reception. Images were post-processed to compensate for the reception profile of the endorectal coil^(13)^.

The protocol included:

- Oblique axial T2W high-resolution 2D FSE MR images (thickness/gap = 3 mm/0 mm; TR/TE = 5600-7400 ms/96-114 ms; ETL = 16; FOV = 180 mm × 180 mm; reconstructed matrix 512 × 512; frequency direction AP, 1 NEX). Acquisition time = 4 min 1 s.
- Axial CUBE T2W 3D FSE MR images (thickness/gap = 1.6 mm/0 mm; TR/TE = 2400 ms/142.4 ms; ETL = 90; FOV = 180 mm × 180 mm; 512 × 512 interpolated matrix in-plane and 2-fold along the craniocaudal axis; frequency direction AP, 2 NEX; flip angle = 90º; receiver bandwidth = 90.91 kHz). Coronal and sagittal reformats were generated. Acquisition time = 4 min 42 s.
- Two 2D single-shot EPI SE high b-value DWI acquisitions (thickness/gap = 3 mm/0 mm; TR/TE = 4725 ms/minimum; FOV = 180 mm × 180 mm; 128 × 64; b-values 0 and 600 and 0 and 1350). Acquisition times = 3 min 52 s and 4 min 29 s. Two ADC maps were reconstructed from each acquisition.
- Oblique axial T1-weighted 3D spoiled gradient echo dynamically contrast enhanced MR images (thickness/gap = 3 mm/0 mm; TR/TE = minimum/minimum; FOV = 260 mm × 260 mm; 192 × 128; 1 NEX; gadopentetate dimeglumine, 0.1 mmol/kg of body weight, at a rate of 3 cm^3^/s using a power injector, followed by a 20 cm^3^ saline bolus at the same rate, 5 min acquisition, temporal resolution = 10 s). Acquisition time = 4 min 58 s.
- 3D ^1^H-MRSI using a water and lipid-suppressed double-spin-echo point resolved spectroscopy sequence (MLEV-PRESS) with spectral-spatial pulses for the two 180º excitation pulses, and outer-voxel saturation pulses (thickness/gap = 3 mm/0 mm; TR/TE = 2000 ms/85 ms; NEX = 1; phase encoding steps = 16 × 10 × 8; FOV = 86 × 54 × 43 mm^3^ yielding a nominal spatial resolution of 0.16 cm^3^). A PRESS volume was selected using the oblique axial T2W images that incorporated the entire prostate while minimizing inclusion of the rectum and peri-prostatic lipids. The PRESS volume was shimmed using an automated phase mapping algorithm, followed by manual shimming of the x, y and z gradients until a water line-width of ≤ 12 Hz was obtained. An interleaved flyback echo-planar spectroscopic readout with a spectral bandwidth of 1012 Hz was used in the left-right dimension. Acquisition time = 7 min 50 s.

The ^1^H-MRSI data were processed using custom processing software^(14)^. The raw data acquired with the modified PRESS incorporating the flyback echo-planar readout trajectory were reordered as previously described^(15)^ and processed in the same manner as the conventional 4D ^1^H-MRSI dataset^(14)^. The spectral data were apodized with a 2-Hz Lorentzian function in the frequency domain, with no filtering in the spatial dimensions. Data were Fourier transformed in the time domain and in three spatial domains. Spectral phase, baseline, and frequency corrections were iteratively made and metabolite peak areas calculated as previously described^(14)^. The 3D ^1^H-MRSI spectral arrays and associated metabolite peak area ratios were overlaid on the corresponding transverse T2W images using the open-source spectral processing package SIVIC.

- Axial T1-weighted FGRE MR images (thickness/gap = 4.2 mm/0 mm; TR/TE = 5.06 ms/2.46 ms; FOV = 240 mm × 240 mm; 192 × 128; NEX = 1). Acquisition time = 2 min 45 s.

### Image interpretation

Two radiologists (8 and 5 years of experience with ^1^H-MRSI and 2 years of experience with PI-RADS v2, i.e. since its initial publication), unaware of the clinical and pathologic data, independently reviewed all scans on a PACS workstation (Impax; Agfa, Mortsel, Belgium) in a single session. To mimic clinical practice, the radiologist could review the T2W, DWI, and DCE sequences in any order, alone or in conjunction. The radiologists had no access to ^1^H-MRSI images at this stage. Up to four suspicious foci were identified and PI-RADS v2 scores assigned to each (Table 1)^(7)^.

**Table 1:** Distribution of imaging findings according to PI-RADS v2 criteria and 1H-MRSI.

	Reader 1			Reader 2	
Sequence	N	(%)		N	(%)
T2W – Peripheral zone					
1 – Uniform hyperintense signal intensity (normal)	0	(0)		0	(0)
2 – Linear or wedge-shaped hypointensity/diffuse mild hypointensity, usually indistinct margin	1	(3.5)		0	(0)
3 – Heterogenous signal intensity or non-circumscribed rounded, moderate hypointensity	11	(37.9)		5	(19.3)
4 – Circumscribed, homogenous moderate hypointense focus/mass confined to prostate and < 1.5 cm in greatest dimension	14	(48.3)		11	(42.3)
5 – As above, but ≥ 1.5 cm in greatest dimension or definitive extra-prostatic extension/invasive behavior	3	(10.3)		10	(38.5)
T2W – Transition zone					
1 – Homogeneous intermediate signal intensity (normal)	0	(0)		0	(0)
2 – Circumscribed hypointense or heterogeneous encapsulated nodule(s) (benign prostatic hyperplasia)	0	(0)		0	(0)
3 – Heterogeneous signal intensity with obscured margins; includes others that do not qualify as 2, 4, or 5	1	(12.5)		1	(10.0)
4 – Lenticular or non-circumscribed, homogeneous, moderately hypointense, and < 1.5 cm in greatest dimension	2	(25.0)		1	(10.0)
5 – As above, but ≥ 1.5 cm in greatest dimension or definite extraprostatic extension/invasive behavior	5	(62.5)		8	(80.0)
DWI					
1 – No abnormality	2	(5.1)		3	(7.7)
2 – Indistinct hypointensity on ADC	0	(0)		0	(0)
3 – Focal mildly/moderately hypointense on ADC and isointense/mildly hyperintense on high b-value DWI	4	(10.3)		8	(20.5)
4 – Focal markedly hypointense on ADC and markedly hyperintense on high b-value DWI; < 1.5 cm in greatest dimension	23	(59.0)		12	(30.8)
5 – As above, but ≥ 1.5 cm in greatest dimension or definite extraprostatic extension/invasive behavior	10	(25.6)		16	(41.0)
DCE					
(–) No early enhancement; or diffuse enhancement not corresponding to a focal finding on T2 and/or DWI; or focal enhancement corresponding to a lesion demonstrating features of benign prostatic hyperplasia on T2WI	11	(28.2)		14	(35.9)
(+) focal, and; earlier than contemporaneously with enhancement of adjacent normal prostate tissue, and; corresponds to suspicious finding on T2W and/or DWI	28	(71.8)		25	(64.1)
Overall score					
1 – Very low (clinically significant cancer is highly unlikely to be present)	0	(0)		0	(0)
2 – Low (clinically significant cancer is unlikely to be present)	2	(5.1)		3	(7.7)
3 – Intermediate (the presence of clinically significant cancer is equivocal)	5	(12.8)		3	(7.7)
4 – High (clinically significant cancer is likely to be present)	23	(59.0)		16	(41.0)
5 – Very high (clinically significant cancer is highly likely to be present)	9	(23.1)		17	(43.6)
^1^H MRSI					
1 – Citrate:choline ratio ≥ 2	3	(7.7)		3	(7.7)
2 – Citrate:choline ratio 1–2	5	(12.8)		8	(20.5)
3 – Choline=citrate	8	(20.5)		12	(30.8)
4 – Choline:citrate ratio 1–2	22	(56.4)		13	(33.3)
5 – Choline:citrate ratio ≥ 2	1	(2.6)		3	(7.7)

N, number of patients.

**Figure 1:**
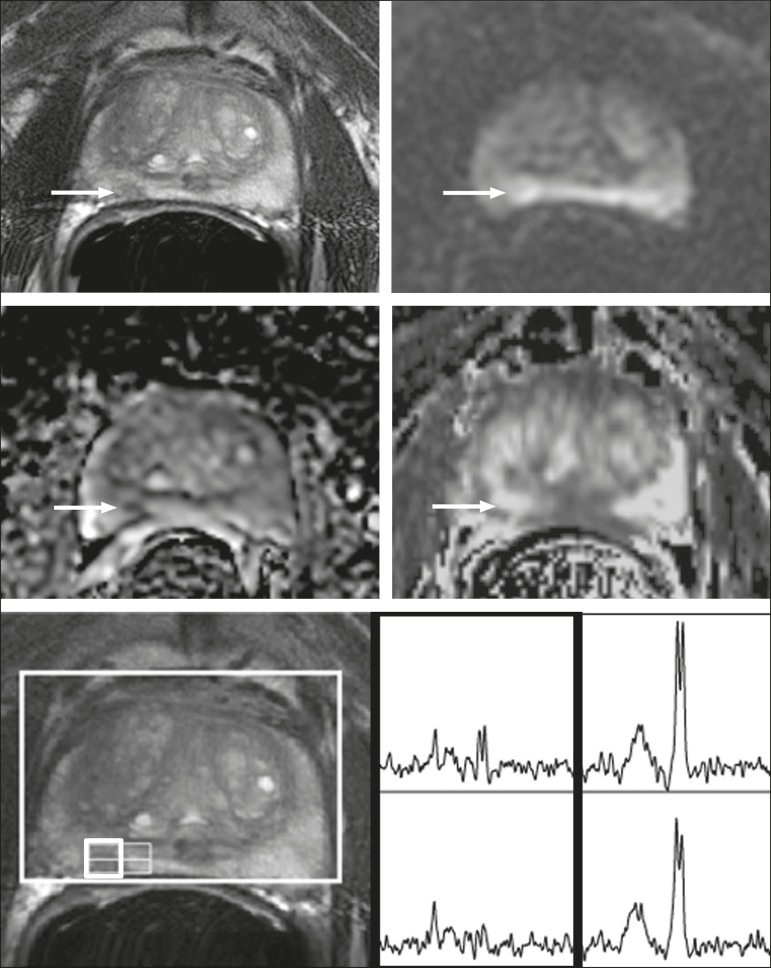
73-year-old man with Gleason 4+3 prostate cancer in the right posterior peripheral zone on radical prostatectomy, corresponding to the findings on imaging (arrows). Reader 1 (and reader 2) characterized the lesion as T2 = 3 (4), DWI = 4 (4), DCE = negative (positive), and overall PI-RADS v2 score = 4 (4). Both readers assigned it a MRSI score of 4 (outlined voxels).

Next the radiologists reviewed the 3D spectral arrays to assign a ^1^H-MRSI score to all suspicious lesions previously assigned a PI-RADS v2 score, i.e. lesions that received a PI-RADS v2 score ranging from 3 to 5. All usable voxels were scored using the five-point scale based on the area ratio of the citrate and choline peaks (Table 1). Figure 1 shows a representative case.

### Surgical technique and histologic evaluation

Experienced urologists performed all radical prostatectomies. Pelvic lymph node dissection was performed based on pre-operative surgical risk. Prostatectomy specimens were marked with ink and fixed overnight in 10% buffered formalin. The glands were sectioned using whole-mount histology at 3 mm intervals in a plane perpendicular to the prostatic urethra. Experienced academic pathologists, unaware of imaging findings, reviewed the histological slides in all cases. The size, location, and Gleason score of all cancer foci seen in the prostate, and the presence, location, and extent of extra-prostatic disease were recorded.

### Statistical analysis

The primary outcomes were the predictions of high-grade PCa, defined as Gleason score ≥ 4+3, and high-stage disease, defined as extra-prostatic extension (EPE) (≥ T3A) at radical prostatectomy on a per patient basis. In the event of multiple lesions, only the index lesion was considered for analyses. The index lesion was defined as the lesion with the highest overall PI-RADS score. If two or more lesions received the same score, the index lesion was the one associated with clear EPE. If none of the lesions demonstrated EPE, the index lesion was the largest one. We assessed the sensitivity, specificity, negative predictive value (NPV), positive predictive value (PPV), and accuracy of the overall PI-RADS v2 score and ^1^H-MRSI score assigned to suspicious lesions for the detection of these outcomes. For high-grade disease, the overall PI-RADS v2 scores 1 to 3 were considered a negative result. For non-organ confined PCa, the overall PI-RADS v2 scores 1 to 4 were considered a negative result. This was because the presence of EPE on mpMRI determines an overall PI-RADS v2 score of 5. For both analyses, ^1^H-MRSI was dichotomized as negative (score ≤ 3) or positive (score 4 or 5).

We compared the areas under the receiver operating characteristic (ROC) curve of univariate logistic regression models that included the overall PI-RADS v2 score or ^1^H-MRSI score; and those to the area under the ROC curve derived from the multivariate models that included the overall PI-RADS v2 and ^1^H-MRSI scores. As mentioned above, if more than one lesion was suspected on mpMRI, only the index lesion was utilized in the analyses.

Interobserver agreement of overall PI-RADS v2 and ^1^H-MRSI scores were calculated utilizing a weighted kappa score (weights = 1 / 1 - 1 / 0.25 - 0.25 - 1 / 0 - 0 - 0.75 - 1 / 0 - 0 - 0.5 - 0.75 - 1).

All analyses were performed using Stata version 13.1 (College Station, TX). *P* values < 0.05 were considered statistically significant.

## RESULTS

The median age was 65 years (interquartile range (IQR), 11). The median PSA at diagnosis was 6.8 ng/mL (IQR, 5.1). CAPRA classified 13 men (33.3%) with low, 21 (53.9%) with intermediate, and 5 (12.8%) with high clinical risk. Thirty-seven men (94.8%) had clinically organ-confined disease at diagnosis; and 28 (71.8%) had biopsy Gleason score ≤ 3+4. The complete clinical, demographic and pathologic characteristics are presented in Table 2.

**Table 2:** Baseline population characteristics.

Variable	Statistic
Age at diagnosis, median (IQR, range)	65 (11, 45–75)
PSA, ng/ml, median (IQR, range)	6.8 (5.1, 1.62–16)
PSA density, median (IQR, range)	0.20 (0.15, 0.06–0.65)
Clinical stage (digital rectal examination), N (%)	
T1c	11 (28.2)
T2a	25 (64.1)
T2b	1 (2.6)
T3a	1 (2.6)
T3b	1 (2.6)
Diagnosis biopsy Gleason grade, N (%)	
3+3	9 (23.1)
3+4	19 (48.7)
4+3	8 (20.5)
4+4	1 (2.6)
5+4	1 (2.6)
5+5	1 (2.6)
Number of diagnostic cores taken, median (IQR, range)	15 (5, 6–24)
Percentage of positive diagnostic cores, median (IQR, range)	25 (29, 8–50)
CAPRA score, %	
0–2 (low risk)	13 (33.3)
3–5 (intermediate risk)	21 (53.9)
6–10 (high risk)	5 (12.8)

IQR, interquartile range; PSA, prostate specific antigen; N, number of patients.

At prostatectomy, 5 men (12.8%) had Gleason score 3+3, 20 (51.3%) had Gleason score 3+4, 12 had Gleason score 4+3 (30.8%), 1 (2.6%) had Gleason score 4+4, and 1 (2.6%) had Gleason score 5+4. Disease was organ-confined (T2C or less) in 19 (48.7%). Twenty men (51.3%) had extra-prostatic extension.

Table 1 reports the complete distribution of imaging findings for both readers; the weighted kappa of the overall PI-RADS v2 and of ^1^H-MRSI scores were 0.62 (95% confidence interval: 0.47-0.81; 88.8% agreement) and 0.46 (95% confidence interval: 0.22-0.70; 76.9% agreement), respectively.

The specificity of ^1^H-MRSI (assigned to a suspicious lesion) to predict Gleason pattern ≥ 4+3 was higher than the specificity of the overall PI-RADS v2 score (56.0%, reader 1, and 68.0%, reader 2, versus 24%, both readers). For the detection of stage ≥ T3a, the use of ^1^H-MRSI scores to further characterize suspicious lesions led, for reader 1, to an increase in sensitivity (64% versus 20%) associated with a decrease in specificity (50% versus 71.4%). No clear differences were seen for reader 2. The performance characteristics are outlined in Table 3.

**Table 3:** Diagnostic test characteristics of PI-RADS v2 and 1H-MRSI for the identification of high grade prostate cancer (dominant Gleason pattern ≥ 4) and extraprostatic disease at radical prostatectomy.

	Sensitivity (95% CI)	Specificity (95% CI)	PPV (95% CI)	NPV (95% CI)
High grade prostate cancer (dominant Gleason pattern ≥ 4)				
		Reader 1		
Overall PI-RADS 2	92.9 (66.1-99.8)	24.0 (9.4-45.1)	40.6 (23.7-59.4)	85.7 (42.1-99.6)
^1^H MRSI	85.7 (57.2-98.2)	56.0 (34.9-75.6)	52.2 (30.6-73.2)	87.5 (61.7-98.4)
		Reader 2		
Overall PI-RADS 2	100.0 (76.8-100)	24.0 (9.4-45.1)	42.4 (25.5-60.8)	100.0 (54.1-100)
^1^H MRSI	57.1 (28.9-82.3)	68.0 (46.5-85.1)	50.0 (24.7-75.3)	73.9 (51.6-89.8)
Extraprostatic disease (stage ≥ T3a)				
		Reader 1		
Overall PI-RADS 2	20.0 (6.8-40.7)	71.4 (41.9-91.6)	55.6 (21.2-86.3)	33.3 (17.3-52.8)
^1^H MRSI	64.0 (42.5-82.0)	50.0 (23.0-77.0)	69.6 (47.1-86.8)	43.8 (19.8-70.1)
		Reader 2		
Overall PI-RADS 2	48.0 (27.8-68.7)	64.3 (35.1-87.2)	70.6 (44.0-89.7)	40.9 (20.7-63.6)
^1^H MRSI	40.0 (21.1-61.3)	57.1 (28.9-82.3)	62.5 (35.4-84.8)	34.8 (16.4-57.3)

95% CI, 95% confidence interval.

**Figure 2:**
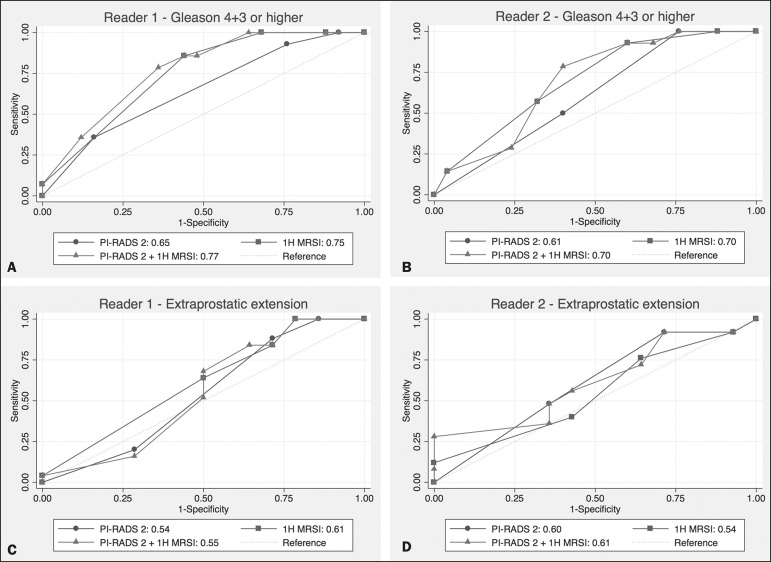
ROC curves for the prediction of pathological Gleason pattern 4 or higher disease (A,B) by the overall PI-RADS v2 score alone and PI-RADS v2 combined with ^1^H-MRSI. Images C and D depict the ROC curves for the prediction of non-organ confined disease (stage T3a or greater) by the same models.

Table 4 details the AUCs for the prediction of Gleason pattern ≥ 4+3 and extraprostatic disease. These results are also illustrated in Figure 2.

**Table 4:** Performance of PI-RADS v2 and 1H-MRSI, alone and combined, for the discrimination of high-grade prostate cancer (dominant Gleason pattern ≥ 4) and extraprostatic disease at radical prostatectomy.

		Reader 1			Reader 2	
Model variables		AUC	95% CI		AUC	95% CI
Dominant Gleason pattern 4 or higher outcome						
Overall PI-RADS 2		0.65	0.49-0.81		0.61	0.45-0.77
^1^H MRSI		0.75	0.62-0.87		0.70	0.55-0.86
Overall PI-RADS 2 + ^1^H MRSI		0.77	0.63-0.91		0.70	0.53-0.86
Pathological stage T3a or higher outcome						
Overall PI-RADS 2		0.54	0.34-0.73		0.60	0.42-0.78
^1^H MRSI		0.61	0.43-0.79		0.54	0.36-0.73
Overall PI-RADS 2 + ^1^H MRSI		0.55	0.34-0.76		0.61	0.43-0.80

95% CI, 95% confidence interval.

Analysis of the shape of the ROC curves shows that the addition of ^1^H-MRSI to PI-RADS v2 improves the prediction of high-grade PCa when lesions are characterized as PI-RADS v2 score 4. There were no statistically significant differences between the AUC of overall PI-RADS v2 (0.65, reader 1; and 0.61, reader 2) and ^1^H-MRSI (0.75, reader 1; and 0.70, reader 2) for either reader. The AUC of overall PI-RADS v2 combined with ^1^H-MRSI was significantly higher than the AUC of overall PI-RADS v2 alone for reader 1 (0.77; *p* = 0.04), but not for reader 2 (0.70; *p* = 0.21).

For the discrimination of stage ≥ T3a, there were no statistically significant differences between the AUC of overall PI-RADS v2 (0.54, reader 1; and 0.60, reader 2), ^1^H-MRSI (0.61, reader 1; and 0.54, reader 2), and their combination (0.55, reader 1; and 0.61, reader 2) for either reader.

## DISCUSSION

Our results suggest that the addition of ^1^H-MRSI to PI-RADS v2 might improve the detection of PCa with Gleason pattern ≥ 4+3, in particular of PI-RADS v2 score 4 lesions; however, it does not seem to increase the detection of high stage (≥ T3a) disease.

Different from its initial version, PI-RADS v2 does not include ^1^H-MRSI. Yet, the PI-RADS Steering Committee encourages “the continued development of promising MRI methodologies”, including ^1^H-MRSI^(16)^, and state that these technologies will be considered for inclusion in future versions, pending new data. While the PI-RADS v2 document does not provide specific reasons for not including ^1^H-MRSI, it is known that it is a complex technique with limited acceptance outside specialized centers due to its long acquisition time, need for local expertise, and general reliance on endorectal coil imaging. Yet, ^1^H-MRSI warrants continue attention; new hardware and software developments may make it more manageable.

Based on previous studies, ^1^H-MRSI improves tumor localization^(17,18)^, volume estimation^(19,20)^, staging^(21)^, tissue characterization^(22)^, and identification of recurrent disease after therapy^(23,24)^. A multicenter study showed that positive MR spectroscopy findings are likely to reflect higher tumor grade and/or volume^(25)^. These studies, though, do have limitations, and there are, also, those with less encouraging results; the ACRIN study published in 2009, for example, showed no difference of accuracy when comparing combined T2W and ^1^H-MRSI and T2W alone^(11)^.

Our results show that the overall PI-RADS v2 score is very sensitive to detect Gleason pattern ≥ 4+3, but its specificity is very low. This suggests PI-RADS v2 is a good option to detect the disease, but not necessarily to characterize it. The use of ^1^H-MRSI, however, led to a 50% increase in specificity, and might at least in some cases help to identify men with high-grade PCa. Our results showed this is particularly true when a lesion receives a PI-RADS v2 score of 4. These results are aligned to those of Giusti et al., who showed that metabolic ratios correlate with Gleason scores^(26)^, and they are similar to those of a meta-analysis in which ^1^H-MRSI had a higher specificity than T2W and increased the specificity of the combination of T2W and DWI^(18)^. While the comparison of overall AUCs (i.e. summary of data for all lesions) found an improvement of discrimination between men with and without PCa Gleason pattern ≥ 4+3 using the combined PI-RADS v2 and ^1^H-MRSI for one reader only, the assessment of the shape of the curves shows a clear separation between the lines of the ROC curves of PI-RADS v2 alone and PI-RADS v2 combined with^1^H-MRSI at the segment that includes only PI-RADS v2 score 4 lesions for both readers. It is possible that this discrepancy in results is due to differences in readers’ experience. ^1^H-MRSI is a complex technique and interpretation can be challenging. It is important to make note of this fact, as these same challenges are likely to be found at other sites that lack radiologist with experience with ^1^H-MRSI.

The metabolic nature of ^1^H-MRSI might explain why it did improve the detection of EPE, as EPE is typically detected on anatomical images. The overall PI-RADS v2 score, however, does include an anatomical assessment. Furthermore, both readers assigned an overall PI-RADS score of 4 or 5 to more than 80% of these suspicious lesions, and an overall PI-RADS v2 score of 5, at least in some instances, characterizes definite EPE^(16)^. An increase in specificity after utilizing ^1^H-MRSI might, therefore, not have been expected. Similarly, because ^1^H-MRSI was applied after the detection of a suspicious lesion using PI-RADS v2, its sensitivity is a direct reflection of this initial detection. Accordingly, it would be expected that positive ^1^H-MRSI results would have a greater impact on lower overall PI-RADS v2 scores. In this study we opted for analyzing only the index lesion, less than 20% of which received a score of 3, likely explaining the lack of benefit of ^1^H-MRSI. It remains, thus, unknown if ^1^H-MRSI would have affected cases presenting with these indeterminate lesions.

We did not find other studies evaluating the incorporation ^1^H-MRSI to PI-RADS v2, but a few authors tested it with its previous version with various results. The studies of Khalifa et al.^(27)^ and Panebianco et al.^(28)^ suggested ^1^H-MRSI improved characterization of PCa and support our results. Yet, the results of Platzek et al.^(29)^ and, more recently, Polanec et al.^(30)^ found that ^1^H-MRSI did not increase the detection and nor improved the grading of PCa. While several possible explanations exist for these discrepancies, the exercise of explaining them is likely not warranted, as the first version of PI-RADS is quite different from PI-RADS v2 and should no longer be utilized. More important, perhaps, is to recognize that considerable interest exists in optimizing the identification high grade or stage disease among men with clinically localized PCa as such determinations may improve management decisions. And that other imaging techniques, including ^1^H-MRSI, may be helpful.

This study has limitations. First, this was a retrospective study with the limitations inherent to this type of design. The population studied was highly selected and included only men who had endorectal mpMRI and radical prostatectomy. We, therefore, probably incurred selection bias and our patients may not fully represent all men with PCa. This is illustrated by the fact most of our lesions were characterized as PI-RADS v2 4 and 5, as men with lower scores are less likely to have cancer and to undergo surgery. However, we considered the need for an adequate standard of reference more important than the limited generalizability. One possible option to prostatectomy is MR-guided biopsy, which can be performed in-bore or by fusion with ultrasound. Accordingly, some of our results, in particular the positive and negative predictive values, do not apply to all men with suspected PCa nor to all men who are under active surveillance and typically have low-grade low-volume disease. Second, we examined endpoints of high-grade and/or non-organ confined disease, but not more distant oncologic endpoints including biochemical recurrence or metastatic progression. Prospective studies with extended follow up may be warranted to definitively evaluate the role of ^1^H-MRSI in improved delineation of PCa outcomes. Third, PI-RADS was designed with the intent to improve the detection of tumors with Gleason score ≥ 3+4, and not ≥ 4+3 as we proposed. This could, perhaps, explain the low specificity of PI-RADS v2 found in this study. More important, though, is that we may have overestimated the diagnostic performance of both PI-RADS v2 and ^1^H-MRSI due to spectrum bias. Spectrum bias refers to the fact that it is usually easier to detect advanced disease than early-stage disease, as subtle abnormalities can be hard to distinguish from normal findings. This typically leads to a higher diagnostic accuracy when a study includes in a population with advanced disease than when the subjects have less severe disease. We opted for characterizing as high-grade tumors only tumors with Gleason score ≥ 4+3 because many institutions consider men with Gleason 3+4 as candidates for active surveillance, while a Gleason score ≥ 4+3 is universally considered an indication for definitive therapy.

In summary, ^1^H-MRSI might improve the discrimination of pathological Gleason score ≥ 4+3 when added to the PI-RADS v2, in particular for lesions that receive a score of 4, but it does not affect the prediction of PCa stage ≥ T3a.
